# Longitudinal associations between gaming and academic motivation during middle childhood

**DOI:** 10.1017/S0033291725101153

**Published:** 2025-08-25

**Authors:** Gabriel Arantes Tiraboschi, Gabrielle Garon-Carrier, Sheri Madigan, Jonathan Smith, Rachel Surprenant, Caroline Fitzpatrick

**Affiliations:** 1 Département d’enseignement au préscolaire et au primaire, Université de Sherbrooke, Sherbrooke, QC, Canada; 2 Département de psychoéducation, Université de Sherbrooke, Sherbrooke, QC, Canada; 3 Department of Psychology, University of Calgary, Calgary, AB, Canada; 4 Alberta Children’s Hospital Research Institute, University of Calgary, Calgary, AB, Canada; 5 Department of Childhood Education, University of Johannesburg, Johannesburg, South Africa

**Keywords:** academic motivation, academic outcomes, random intercept cross-lagged panel model, structural equation modelling, school-aged children, middle childhood, boys, sex differences, screen use, media use, video game playing, gaming

## Abstract

**Background:**

Child video game playing (“gaming”) may lead to decreased child academic motivation. Conversely, children with low academic motivation may seek fulfillment through gaming. We examined bidirectional associations between child gaming and academic motivation across middle childhood.

**Methods:**

Our analyses are based on 1,631 children (boys = 785) followed in the context of the Quebec Longitudinal Study of Child Development. Data on gaming and academic motivation were collected repeatedly at ages 7, 8, and 10. Measures of child gaming were parent-reported and reflect daily video game playing time. Measures of academic motivation were child self-reported and reflect enjoyment in learning mathematics, reading, and writing. To disentangle the directionality of associations, we estimated a random-intercept cross-lagged panel model to estimate bidirectional, within-person associations between gaming and academic motivation in a cohort of school-aged Canadian children.

**Results:**

Our results revealed unidirectional associations whereby more frequent gaming by boys at age 7 years predicted lower academic motivation at age 8 years (*β* = −.11, 95% confidence interval [CI]: −.22 to −.01), and similarly, gaming by boys at age 8 years predicted lower academic motivation at age 10 years (*β* = −.10, 95% CI: −.19 to −.01). Changes in boys’ academic motivation did not contribute to subsequent changes in gaming. There were no associations between gaming and academic motivation for girls.

**Conclusions:**

More time devoted to gaming among school-aged boys is associated with reduced academic motivation during a critical developmental period for the development of academic skills. Fostering healthy gaming habits may help promote academic motivation and success.

Video game playing (“gaming”) has long been a popular activity, especially among children and adolescents (Ivory, 2015). According to a Canadian survey, 88–95% of children aged 6 and 12 years play video games, with girls playing 9 h and boys 12 h per week on average (Entertainment Software Association of Canada, 2020). Another survey conducted in the United States indicates that the average daily gaming time for children aged 4–8 years is 40 min, and this duration increases with age (Rideout & Robb, 2020). However, high levels of gaming among children have prompted concerns because video games are intentionally designed to be as engaging and rewarding as possible (Hodent, 2017), with some even incorporating *gamblification* features (Király et al., 2022). As an example, a growing trend in video game design, introduced in 2003, known as “loot boxes,” significantly enhances player engagement and spending by employing randomized reward mechanisms akin to gambling (Lemmens, 2022; Spicer et al., 2022; Zendle et al., 2020). This may then pose a significant risk for the developing reward systems of young players (Spicer et al., 2022; Zendle et al., 2019).

According to studies conducted with adults, the intensity of striatal dopamine released during gaming is comparable to the effect of substances such as alcohol and stimulants (Boileau et al., 2003, 2007; Cox et al., 2009; Koepp et al., 1998). Consistent with the activation of reward-related pathways, high engagement in gaming can lead players to experience cravings for gaming (Antons et al., 2023). Supporting this, there is also evidence that gaming is associated with neuroplastic changes in brain regions involved in reward processing (Kühn et al., 2011; Palaus et al., 2017). Moreover, in addition to external rewards, it has been proposed that gaming can provide a sense of competence, autonomy, and social connectedness, which are key elements of intrinsic motivation (Przybylski et al., 2010). Coupled with compelling mechanisms designed to maintain maximum engagement (e.g. Király et al., 2022), these elements make gaming highly appealing and a difficult habit to regulate, especially for children, whose developing self-regulatory abilities may heighten their vulnerability to its allure and addictive qualities.

Given its high level of stimulation and engagement, more frequent gaming by children may lead to decreased enjoyment and motivation for other, more mundane activities (Christodoulou et al., 2020; Klimczyk, 2023), such as academic learning. According to a large-scale cross-sectional study of adolescents, gaming for more than 2 h/day was significantly associated with reduced time devoted to homework and a higher incidence of school absenteeism (Hellström et al., 2012). Similarly, another large-scale cross-sectional study found that children and adolescents who game spent less time reading and studying compared to non-gamers (Cummings & Vandewater, 2007). This study estimated that for each hour boys spent gaming on weekdays, they spent 2 min less reading (30% decrease), and every hour girls played video games during weekdays was associated with 13 min less doing homework (34% decrease). Yet, despite evidence associating gaming with lower academic engagement, the possible underlying mechanisms – such as diminished academic motivation – remain insufficiently investigated.

The school-age years represent a crucial time for children to cement key academic skills that will pave the way for their educational attainment and healthy development (Allison, et al., 2019; Peng & Kievit, 2020; Williams et al., 2022). Thus, it remains crucial to investigate how gaming, a common leisure activity among children, may impact academic motivation at this age. Understanding the directionality of such an association is equally crucial, as past research on the associations between gaming and educational outcomes has been predominantly cross-sectional (Adelantado-Renau et al., 2019). Overcoming this limitation is relevant because video games may either influence academic motivation or act as an alternative source of fulfillment for students who experience low levels of enjoyment at school. Indeed, past correlational research has shown that higher levels of obsessive gaming were correlated with lower levels of overall life satisfaction and basic psychological need satisfaction (Przybylski et al., 2009).

In light of the limitations in the literature, in the current study, we test bidirectional associations between child gaming and academic motivation at ages 7, 8, and 10 years using a random-intercept cross-lagged panel model (RI-CLPM) (Hamaker et al., 2015). An important strength of this statistical model is that it models within-person changes. In other words, each person serves as their own baseline control, which accounts for stable, existing individual differences in social, psychological, and academic experiences (e.g. socioeconomic differences). Since gaming and academic motivation differ between boys and girls (Hastings et al., 2009; Leonhardt & Overå, 2021; Niskier et al., 2024), we estimate associations separately for boys and girls.

## Methods

### Participants and setting

Participants were part of the Quebec Longitudinal Study of Child Development (QLSCD, 1998–2023), a population-based cohort intended to study child biopsychosocial development and academic adjustment. For the QLSCD, birth registers were used to create a stratified random sample of families with infants born between 1997 and 1998 in the province of Quebec (Canada). Inclusion criteria included pregnancy lasting between 24 and 42 weeks and mothers being proficient in either French or English. In total, 2,120 participating families were followed longitudinally and subsequently assessed yearly or biennially. The current study includes data from the QLSCD collected in 2005, 2006, and 2008, when children were at ages 7 (*n* = 1,537), 8 (*n* = 1,526), and 10 (*n* = 1,402) years. Participating children with information on gaming and academic motivation, at least at one time point, were retained in the current study. Parents of participating children provided informed consent, and children also provided assent at age 10 years. The Health Quebec ethics committee approved the QLSCD protocol. Additional details on the QLSCD are described elsewhere (Orri et al., 2021).

## Measures

### Gaming (video game playing time)

At ages 7, 8, and 10, parents reported child gaming responding to the following question: “On average, how much time does your child spend each day playing computer or video games?.” Responses included: “None,” “Less than one hour,” “From 1 up to 3,” “From 3 up to 5,” “From 5 up to 7,” “More than 7 hours.” These categories were converted as follows: 0, 0.5, 2, 4, 6, and 8 h/day. This question was developed for the QLSCD and can be accessed at https://www.jesuisjeserai.stat.gouv.qc.ca/informations_chercheurs/outils_collecte/outils_collecte_an.html.

### Academic motivation

At ages 7, 8, and 10, children were asked by a trained interviewer about their interest in reading, writing, and mathematics with the following questions – 1: “I like *reading/writing/math*,” 2: “*Reading/writing/math* interest me a lot,” 3: “I *read/write/do math* even when I am not obliged to do so.” Their responses were reported on a 5-point Likert scale ranging from “ *Always no* ” to “**Always yes*.*” Child responses to these nine questions were then used to estimate a standardized global intrinsic motivation score ranging from 0 to 10. Our measure was derived from the intrinsic motivation subscale of the Elementary School Motivation Scale, which is based on the self-determination theory of motivation (Guay et al., 2010; Ryan, 2023). Internal reliability was adequate, *α* = .72–.80. Furthermore, this measure has been previously validated and is a robust predictor of academic engagement and achievement (Garon-Carrier et al., 2016).

### Statistical analysis

To estimate the direction of associations between gaming and academic motivation in school-aged children, we use a RI-CLPM (Hamaker et al., 2015). The RI-CLPM is a structural equation model used to determine the temporal sequencing of associations between two variables that are measured repeatedly across time. The RI-CLPM has the advantage of separating stable between-person effects (e.g. individual differences in academic motivation due to stable factors and socioeconomic status) from dynamic within-person effects (e.g. how children’s deviations from their own baseline of video game consumption in 1 year affect their academic motivation 1 year later relative to their unique baseline). In a RI-CLPM, the cross-lagged and autoregressive paths measure only within-person fluctuations. More information on the RI-CLPM is available elsewhere (Baribeau et al., 2022; Hamaker et al., 2015; Mulder & Hamaker, 2021). Recommended cutoff points to interpret the effect sizes of cross-lagged paths are <.07 (small), .07–.11 (medium), and ≥.12 (large) (Orth et al., 2022).

**Figure 1.**
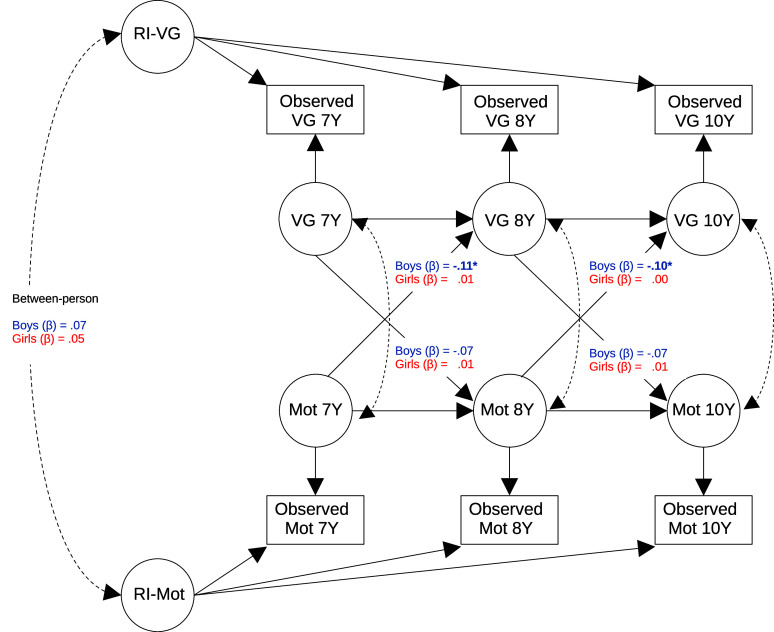
Random-intercept cross-lagged panel model of academic motivation and video game playing between ages 7 and 10 . Each shape represents a variable. Circles are latent variables, and rectangles are observable variables. Straight arrows represent regressions, and curved arrows represent covariances. Asterisks indicate significant associations (*p* < .05). Indicated in the picture are the standardized estimates of the cross-lagged within-person effects and the between-person associations. Factor loadings of random intercepts were constrained to 1.00. Mot, academic motivation; RI, random-intercept latent variable; Observed, observed variables at data collection; VG, video game playing levels; Y, age in years. Data compiled from the final master file of the Québec Longitudinal Study of Child Development (1998–2023), ©Gouvernement du Québec, Institut de la statistique du Québec, Canada.

Before specifying our model, based on our a priori hypothesis that the results would be different between boys and girls, we first checked if the variances and covariances of gaming and academic motivation variables were sex-invariant. We compared the model fit of a matrix freely estimated with a matrix constrained for equality across sexes, using multigroup analysis. A significant *χ* ^2^ implies that the hypothesis of equal variance/covariance structures for boys and girls can be rejected, which would provide additional empirical support to stratify our model by the sex of the children. Subsequently, we compared the fit of a model constraining cross-lagged regressions for equality over time with the fit of a freely estimated model (Mulder & Hamaker, 2021). The model was defined after testing the invariance between sex and cross-lagged paths over time. In terms of assumptions, our sample size was sufficient (*n* = 1,631 and *n* > 20 per observable variable), and we observed no extreme collinearities (all correlations *r* < .80). However, child gaming variables were not normally distributed (skewness > |2| and kurtosis > |7|). For this reason, we estimated our model using the Huber–White robust variant of the maximum likelihood (ML) estimator, which accommodates non-normal distributions. We estimated the final model by including repeated measures of gaming time and academic motivation at ages 7, 8, and 10 years. See Figure 1 for a representation of our model. The R code for the model specification is provided in the Supplementary Materials. The full model formula for the RI-CLPM is specified elsewhere (Hamaker et al., 2015).

### Missing data

Missing data from ages 7 to 10 years ranged from 19.3 to 22.6% for gaming and 9.5 to 19.2% for academic motivation. Across all time points, the proportion of full respondents (participants with data at all time points) was 62.4% for gaming and 73.6% for motivation, the proportion of partial respondents was 32.9% for gaming and 24.2% for motivation, and the proportion of nonrespondents was 4.7% for gaming and 2.3% for motivation. Little’s test revealed that the data are not missing completely at random (*χ* ^2^ = 277, df = 148, *p* < .001). Given the characteristics of the missing data, and in line with Newman (2014), we opted to use full information ML to address missing data.

## Results

### Descriptive statistics

Of the 1,631 children, 48.1% (*n* = 785) were boys and 51.9% (*n* = 846) were girls. Data on gaming time or academic motivation were available for 1,514 children (boys = 721) at age 7 years, 1,509 children (boys = 719) at age 8 years, and 1,392 children (boys = 670) at age 10 years. At all time points, boys spent significantly more time playing video games than girls (mean difference = .413 h/day, 95% confidence interval [CI] = .347 to .479), and girls had significantly higher academic motivation compared to boys (mean difference = .398, 95% CI = .242 to .553). Gaming increased significantly from ages 7 to 10 years (mean difference = .214 h/day, 95% CI = .071 to .250) for both sexes. Conversely, academic motivation significantly decreased from age 7 to 10 years (mean difference = −.287, 95% CI = −.414 to −.159) for boys and girls. Descriptive statistics are summarized in Table 1.

**Table 1.** Descriptive statistics for child gaming and academic motivation

					Data distribution		Welch’s *t*-test
	SEX	*N*	Missing	Mean	SD	IQR	*p*-value
VG age 7	Boys	629	156	1.084	1.143	1.500	
	Girls	688	158	0.598	0.638	0.000	<.001
Mot age 7	Boys	701	84	7.291	1.969	3.000	
	Girls	775	71	7.578	1.759	2.600	0.003
VG age 8	Boys	597	188	1.061	0.854	0.429	
	Girls	666	180	0.614	0.511	0.571	<.001
Mot age 8	Boys	698	87	6.957	2.098	2.600	
	Girls	769	77	7.367	1.771	2.200	<.001
VG age 10	Boys	632	153	1.083	0.900	0.946	
	Girls	697	149	0.747	0.579	0.429	<.001
Mot age 10	Boys	621	164	6.330	1.725	2.600	
	Girls	697	149	6.862	1.732	2.500	<.001

*Note:* Descriptive statistics of hours per day of video game use and academic motivation of boys and girls between ages 7 and 8 years. IQR, interquartile range; Mot, academic motivation in writing, reading, and mathematics ranging from 0 to 10; SD, standard deviation; VG, video game playing in hours/day. Welch’s *t*-test was used to test differences between boys’ and girls’ means. Data compiled from the final master file of the Québec Longitudinal Study of Child Development (1998–2023), ©Gouvernement du Québec, Institut de la statistique du Québec, Canada.

### Invariance testing

The fit comparison of the freely estimated and the constrained variance/covariance matrix of boys and girls revealed a significant difference between models (Δ *χ* ^2^ = 213.46, Δdf = 15, *p* < .001), suggesting that relaxing constraints between boys and girls significantly improves the model. This result corroborates our decision to stratify our analysis by sex. We then compared a time-invariant model with a freely estimated model over time, which yielded no significant differences (Δ *χ* ^2^ = 6.3, Δdf = 8, *p* = .61), suggesting that the lagged effects are time-invariant (Mulder & Hamaker, 2021). Therefore, we decided to constrain the lagged effects over time in our model.

### Random-intercept cross-lagged panel model

Our RI-CLPM generated excellent global fit indices (root mean square error of approximation [RMSEA] = 0.000, standardized root mean square residual [SRMR] = 0.018, Robust comparative fit index [CFI] = 1.000, and *χ* ^2^ = 7.712, df = 10, *p* = .657) and local fit indices (all standardized residuals ≤ |1.96|; see Supplementary Materials). A total of 52 free parameters were estimated in the model, yielding a ratio of 5.2 relative to the degrees of freedom. Our model is represented in Figure 1 along with the results of cross-lagged effects. The full results of our model are described in Table 2, including the autoregressive effects, cross-lagged effects, and between-person associations. Most relevant to our hypothesis are the cross-lagged effects. For boys, within-person level increases in gaming predicted lower levels of academic motivation from ages 7 to 8 (*β* = −.117, 95% CI = −.226 to −.008), and from ages 8 to 10 (*β* = −.105, 95% CI = −.197 to −.014). This suggests a medium to large effect of gaming on academic motivation for boys between the ages of 7 and 10 (Orth et al., 2022). Conversely, academic motivation did not predict boys’ later levels of gaming from ages 7 to 8 years (*β* = −.074, 95% CI = −.192 to .044), nor from age 8 to 10 (*β* = −.075, 95% CI = −.190 to .041).

**Table 2.** RI-CLPM longitudinal relationship between child gaming and academic motivation for boys and girls separately

				95% Confidence interval (*β*)		Two-tailed
Regression path	Sex	*B*	*β*	Lower	Upper	*p*-values
VG age 7 → Mot age 8	Boys*	−.210	−.117	−.226	−.008	.026
	Girls	.032	.012	−.091	.115	.818
Mot age 7 → VG age 8	Boys	−.032	−.074	−.192	.044	.215
	Girls	.005	.017	−.096	.130	.767
VG age 8 → Mot age 10	Boys*	−.210	−.105	−.197	−.014	.026
	Girls	.032	.009	−.069	.087	.818
Mot age 8 → VG age 10	Boys	−.032	−.075	−.190	.041	.215
	Girls	.005	.015	−.082	.111	.767
VG age 7 → VG age 8	Boys	.103	.145	−.031	.321	.130
	Girls	.088	.120	−.095	.334	.295
VG age 8 → VG age 10	Boys	.103	.097	−.029	.223	.130
	Girls	.088	.074	−.074	.223	.295
Mot age 7 → Mot age 8	Boys*	.188	.175	.072	.279	.001
	Girls*	.160	.158	.044	.273	.008
Mot age 8 → Mot age 10	Boys*	.188	.238	.100	.377	.001
	Girls*	.160	.166	.040	.291	.008
Between-person covariance	Boys	.026	.074	−.363	.510	.731
(RI-VG ↔ RI-Mot)	Girls	.013	.056	−.244	.356	.712

*Note*: Results from the random-intercept cross-lagged panel model (RI-CLPM) divided by sex, including cross-lagged paths, autoregressive paths, and between-person associations, respectively. *B* represents the unstandardized estimate of each regression path. *β* represents the standardized estimate of the regression, followed by the 95% confidence interval of *β. P*-values < 0.05 were considered significant (marked with *). Mot, academic motivation in writing, reading, and mathematics; VG, video game playing. Data compiled from the final master file of the Québec Longitudinal Study of Child Development (1998–2023), ©Gouvernement du Québec, Institut de la statistique du Québec, Canada.

For girls, no cross-lagged effects were significant. Video game playing did not predict subsequent academic motivation from ages 7 to 8 years (*β* = .012, 95% CI = −.091 to .115), and from ages 8 to 10 years (*β* = .009, 95% CI = −.069 to .087). Similarly, academic motivation did not predict gaming from ages 7 to 8 years (*β* = .017, 95% CI = −.096 to .130) and from ages 8 to 10 years (*β* = .015, 95% CI = −.082 to .111). For both boys and girls, we found no significant between-person associations between gaming and academic motivation.

### Sensitivity analyses

We also estimated a RI-CLPM using complete cases only to assess the potential bias of our missing data approach. This model generated excellent fit indices as well (RMSEA = 0.000, SRMR = 0.019, Robust CFI = 1.000, and *χ* ^2^ = 8.124, df = 10, *p* = .617). A similar pattern of results was observed, suggesting little to no bias. There were no significant within-person cross-lagged effects for girls. For boys, within-person increases in gaming predicted lower levels of academic motivation from ages 7 to 8 years (*β* = −.131, 95% CI = −.231 to −.030), and from ages 8 to 10 years (*β* = −.103, 95% CI = −.179 to −.028). Cross-lagged paths from motivation to subsequent gaming were all nonsignificant. Full results of the model using complete cases are available in the Supplementary Materials.

One limitation of the RI-CLPM is that it does not provide information on between-person effects longitudinally. To assess how between-person effects affect our model longitudinally, we performed a traditional cross-lagged panel model (CLPM) to compare results with our RI-CLPM. The CLPM presented good fit indices (RMSEA = 0.05, SRMR = 0.041, Robust CFI = .929, and *χ* ^2^ = 48.5, df = 16, *p* = .000). In terms of our main hypothesis, the results lead to the same conclusions: gaming predicted lower levels of academic motivation from ages 7 to 8 years (*β* = −.074, 95% CI = −.138 to −.009), and from ages 8 to 10 years (*β* = −.067, 95% CI = −.125 to −.010) among boys but not girls. Full results of the CLPM are available in the Supplementary Materials. Because CLPMs are nested in RI-CLPMs, we also performed a difference *χ* ^2^-test statistic to compare the fit of the two models. The comparison revealed a significantly better fit in the RI-CLPM (ΔAkaike Information Criterion = 36, ΔBayesian Information Criterion = 3, Δ *χ* ^2^ = 41.2, Δdf = 6, *p* = .000).

Finally, we calculated intraclass correlation (ICC) for both gaming and academic motivation to disentangle the proportion of variance explained by between-person differences from within-person fluctuations. ICC values can be interpreted as the proportion of variance that is explained by between-person rather than within-person variation. Gaming ICC_3,1_ was 0.307 (95% CI = .276 to .338) and motivation ICC_3,1_ was 0.333 (95% CI = .302 to .364), both indicating high longitudinal within-person variability, which accounts for more than 60% of the variance, and further suggesting that a within-person change model was warranted.

## Discussion

To our knowledge, the present study is the first to provide longitudinal evidence that higher levels of child gaming are associated with reduced academic motivation among school-aged boys. The majority of previous studies on the effects of gaming on academic outcomes have used cross-sectional designs or have been unable to account for the direction of association (Adelantado-Renau et al., 2019; Mundy et al., 2020; Sanders et al., 2019; Sharif et al., 2010). Our results help clarify the directionality and temporality of these associations by showing that higher levels of gaming in boys predict lower levels of academic motivation, and not vice versa. Our observed effect sizes ranged from medium to large, which indicates meaningful or clinically significant effects of gaming on boys’ academic motivation during middle childhood (Orth et al., 2022). Finally, associations were observed for boys, but not for girls.

Our results expand previous research suggesting that gaming among children and adolescents is linked to attentional difficulties, reduced academic engagement, and lower academic achievement (Adelantado-Renau et al., 2019; Hellström et al., 2012; Tiraboschi et al., 2022). Directional associations from gaming to academic motivation were only observed in boys in our study. The sex-specific nature of our findings may be due to boys’ tendency to spend more time playing video games than girls (Leonhardt & Overå, 2021). As such, increased gaming time among boys might encroach upon time otherwise allocated to academic endeavors (Cummings & Vandewater, 2007). In addition, boys and girls also differ in the types of video games they play, with boys tending to play more competitive multiplayer and violent video games (Hastings et al., 2009). As such, the types of video games played by boys may be more detrimental to their development and eventual academic motivation (Eshuis et al., 2023; Vargas-Iglesias, 2020; West et al., 2018).

The observed association between child gaming and academic motivation highlights the need for further research on the underlying mechanisms that may explain this relationship. One possibility is that video game reward schedules could alter child reward sensitivity, making them less responsive to incentives offered in the school context (Drummond & Sauer, 2018; Hodent, 2017; Li et al., 2019). According to one longitudinal study, the association between child screen use (including gaming and TV) at age 10 years and school performance at age 14 years is mediated by child sensation-seeking behavior (Sharif et al., 2010). This suggests that child gaming may impact academic performance by diminishing motivation for more effortful and less novel activities. Another possibility is that associations between gaming and academic motivation are accounted for by increased symptoms of hyperactivity and inattention, which could result from increased gaming and contribute to lower academic motivation (Masi et al., 2021; Paulus et al., 2018; Smith et al., 2020; Tiraboschi et al., 2022). Finally, as gaming duration increases, the amount of time children spend studying likely decreases (Cummings & Vandewater, 2007), which could undermine their academic skills and, consequently, their academic motivation (Hartanto et al., 2018; Schaffner et al., 2016).

In terms of practical implications and recommendations, our results suggest that parents be sensitized about gaming and its consequences during middle childhood. In addition, practitioners can be encouraged to offer information to parents about the potential consequences of gaming for academic and behavioral outcomes, particularly for boys. Furthermore, practitioners can encourage families to develop a personalized family media use plan, paying particular attention to gaming during middle childhood (HealthyChildren.org, 2024).

In terms of strengths, our study is the first to demonstrate that video game playing predicts lower levels of academic motivation in school-aged boys, and not vice versa. As such, an important strength of our study is our ability to disentangle the direction of this association. Furthermore, specifically estimated associations at the within-person level provide a robust statistical control for stable individual (i.e. baseline motivation and academic competence) and environmental (i.e. socioeconomic status and school environment) factors. Finally, our study is strengthened by using a large population-based sample, which strengthens the generalizability of our findings.

Our study is not without limitations. Our observational design prevents us from discarding some potential time-varying confounders, such as child or parent mental health (Gubbels et al., 2019). Therefore, no causal conclusions should be drawn, as a third variable associated with both gaming and academic motivation could explain the observed association. Randomized controlled experiments are needed to confirm a causal effect of child gaming on academic motivation. Another limitation is that the use of parent reports of child gaming may be subject to recall and social desirability bias. Nonetheless, our incorporation of child-reported measures of academic motivation helps reduce the possibility of shared measurement bias. Our results are also limited by our focus on the amount of time children spent playing video games, without consideration of video game content or gameplay features. Furthermore, in the present study, we measured intrinsic motivation specifically. As such, future studies could examine associations between child gaming and additional dimensions of motivation and academic outcomes, such as school engagement, connectedness, and academic achievement. A final limitation is that our data were collected between 2005 and 2008. As such, replications are warranted with more recent longitudinal data.

Future studies should examine possible moderators of the observed association. In particular, the characteristics of games and child gameplaying warrant particular attention. For instance, frequent engagement in multiplayer gaming may lead to more adverse outcomes than more casual single-player gaming (Przybylski & Mishkin, 2016). Similarly, video games featuring gamblification elements (e.g. loot boxes) or employing more intense reward schedules may exert a stronger disruptive effect on academic motivation (Antons et al., 2023). Patterns of gameplay are also important to consider. For example, gaming on weekdays may have different implications for academic motivation than gaming primarily on weekends (Hartanto et al., 2018). Finally, studies could also employ person-centered approaches to help identify subgroups of children who may be more vulnerable to the negative consequences of video game playing on academic outcomes. Indeed, according to one cross-sectional study of 12-year-olds, heavy gamers displayed lower academic engagement when compared to non-gamers, but no associations with academic engagement were found for those who played <3 h/day (Przybylski & Mishkin, 2016). As such, person-centered approaches may be useful for identifying children who may be more vulnerable to the negative consequences of video game playing.

## Conclusion

Child gaming is widespread among children and adolescents. Our study provides compelling evidence that more time devoted to this common leisure activity is associated with lower academic motivation during the school-aged years. Notably, the influence of gaming on academic motivation appears more pronounced in boys, potentially reflecting gender differences in gaming interests and habits. Together, these findings indicate that parents, healthcare professionals, and educators should be encouraged to monitor child gaming, particularly for boys in the early years of elementary school.

## Supporting information

Tiraboschi et al. supplementary material Tiraboschi et al. supplementary material

## Supplementary material

The supplementary material for this article can be found at http://doi.org/10.1017/S0033291725101153.
