# Longitudinal associations between gaming and academic motivation during middle childhood – CORRIGENDUM

**DOI:** 10.1017/S0033291725103000

**Published:** 2026-02-19

**Authors:** Gabriel Arantes Tiraboschi, Gabrielle Garon-Carrier, Sheri Madigan, Jonathan Smith, Rachel Surprenant, Caroline Fitzpatrick

**Affiliations:** 1 Département d’enseignement au préscolaire et au primaire, Université de Sherbrooke, Sherbrooke, QC, Canada; 2 Département de psychoéducation, Université de Sherbrooke, Sherbrooke, QC, Canada; 3 Department of Psychology, University of Calgary, Calgary, AB, Canada; 4 Alberta Children’s Hospital Research Institute, University of Calgary, Calgary, AB, Canada; 5 Department of Childhood Education, University of Johannesburg, Johannesburg, South Africa

When this article was originally published in Psychological Medicine it contained an error with Figure 1. The correct version can be found below:

**Figure 1.**
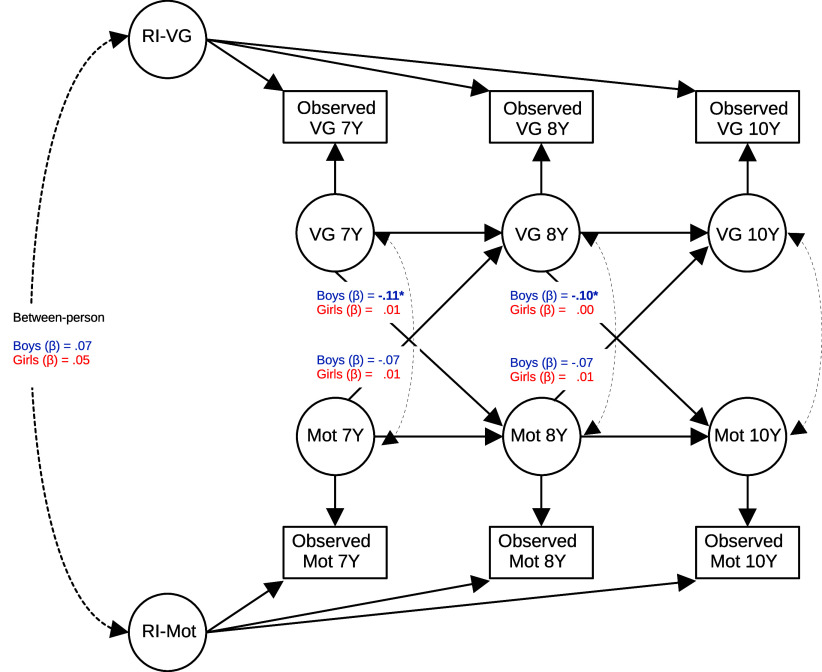
Random-intercept cross-lagged panel model of academic motivation and video game playing between ages 7 and 10. Each shape represents a variable. Circles are latent variables, and rectangles are observable variables. Straight arrows represent regressions, and curved arrows represent covariances. Asterisks indicate significant associations (*p* < .05). Indicated in the picture are the standardized estimates of the cross-lagged within-person effects and the between-person associations. Factor loadings of random intercepts were constrained to 1.00. Mot, academic motivation; RI, random-intercept latent variable; Observed, observed variables at data collection; VG, video game playing levels; Y, age in years. Data compiled from the final master file of the Québec Longitudinal Study of Child Development (1998–2023), ©Gouvernement du Québec, Institut de la statistique du Québec, Canada.

The authors apologise for this error.
