# Supplementary material for: Longitudinal associations between gaming and academic motivation during middle childhood
Source: Psychol Med. 2025 Aug 25;55:e235. doi: 10.1017/S0033291725101153 (PMC12404323; doi:10.1017/S0033291725101153)
Supplement: Tiraboschi et al. supplementary material [file S0033291725101153sup001.docx]

**Supplementary Content**

| **Table 1. Results from the lagged effects of the RI-CLPM using the listwise deletion method** | | | | | | | | | | | | |
| --- | --- | --- | --- | --- | --- | --- | --- | --- | --- | --- | --- | --- |
|  | | | | | | | **95% Confidence Interval (β)** | | | | **Two-tailed** | |
| **Regression path** | **Sex** | | ***B*** | | **β** | | **Lower** | | **Upper** | | **p-values** | |
| VG age 7 → Mot age 8 | | Boys* | -.224 |  | -.131 |  | -.231 |  | -.030 |  | .008 |  |
|  | | Girls | .044 |  | .015 |  | -.095 |  | .126 |  | .784 |  |
| Mot age 7 → VG age 8 | | Boys | -.035 |  | -.090 |  | -.220 |  | .041 |  | .181 |  |
|  | | Girls | .011 |  | .046 |  | -.088 |  | .181 |  | .497 |  |
| VG age 8 → Mot age 10 | | Boys* | -.224 |  | -.103 |  | -.179 |  | -.028 |  | .008 |  |
|  | | Girls | .044 |  | .011 |  | -.069 |  | .092 |  | .784 |  |
| Mot age 8 → VG age 10 | | Boys | -.035 |  | -.087 |  | -.211 |  | .036 |  | .181 |  |
|  | | Girls | .011 |  | .039 |  | -.073 |  | .150 |  | .497 |  |
| VG age 7 → VG age 8 | | Boys | .108 |  | .166 |  | .014 |  | .318 |  | .072 |  |
|  | | Girls | .070 |  | .104 |  | -.121 |  | .328 |  | .372 |  |
| VG age 8 → VG age 10 | | Boys | .108 |  | .102 |  | -.015 |  | .219 |  | .072 |  |
|  | | Girls | .070 |  | .055 |  | -.073 |  | .184 |  | .372 |  |
| Mot age 7 → Mot age 8 | | Boys* | .245 |  | .236 |  | .106 |  | .366 |  | .000 |  |
|  | | Girls* | .228 |  | .217 |  | .096 |  | .339 |  | .001 |  |
| Mot age 8 → Mot age 10 | | Boys* | .245 |  | .298 |  | .150 |  | .446 |  | .000 |  |
|  | | Girls* | .228 |  | .247 |  | .104 |  | .390 |  | .001 |  |
| Between-person covariance | | Boys | .014 |  | .052 |  | -.507 |  | .611 |  | .851 |  |
| (RI-VG ↔ RI-Mot) | | Girls | .012 |  | .060 |  | -.320 |  | .440 |  | .758 |  |
| Note. Results from the Random Intercept Cross-Lagged panel model (RI-CLPM) divided by sex, including cross-lagged paths, autoregressive paths, and between-person associations, respectively. *B* represents the unstandardized estimate of each regression path. β represents the standardized estimate of the regression, followed by the 95 % confidence interval of β. P-values lower than 0.05 were considered significant (marked with *). VG = Video Game use and Mot = Academic motivation in writing, reading, and math. | | | | | | | | | | | | |
|  | | | | | | | | | | | | |

**Table 2. Standardized residuals for boys’ model (RI-CLPM using FIML for missing data)**

|  | VG age 7 | VG age 8 | VG age 10 | Mot age 7 | Mot age 8 | Mot age 10 |
| --- | --- | --- | --- | --- | --- | --- |
| VG age 7 | 0.665 |  |  |  |  |  |
| VG age 8 | 0.648 | -0.185 |  |  |  |  |
| VG age 10 | 0.932 | -0.500 | -0.241 |  |  |  |
| Mot age 7 | 0.027 | 1.615 | -1.150 | 0.263 |  |  |
| Mot age 8 | -1.377 | -0.070 | -1.741 | 0.206 | 0.518 |  |
| Mot age 10 | -0.253 | 1.461 | -0.701 | -0.612 | -0.346 | -0.537 |
| Mean residuals | 0.617 | -1.669 | -0.374 | -1.140 | -1.425 | 1.049 |
| Note. Residuals of variances and covariances between variables and residuals for the mean estimates of individual variables. All residuals are standardized. VG = Video Game use and Mot = Academic motivation in writing, reading, and math. | | | | | | |

**Table 3. Standardized residuals for girls’ model (RI-CLPM using FIML for missing data)**

|  | VG age 7 | VG age 8 | VG age 10 | Mot age 7 | Mot age 8 | Mot age 10 |
| --- | --- | --- | --- | --- | --- | --- |
| VG age 7 | -0.937 |  |  |  |  |  |
| VG age 8 | -1.037 | 1.531 |  |  |  |  |
| VG age 10 | -1.034 | 1.038 | 1.449 |  |  |  |
| Mot age 7 | -0.161 | 0.383 | 1.097 | 0.028 |  |  |
| Mot age 8 | -0.400 | -0.223 | -0.062 | -0.093 | -0.121 |  |
| Mot age 10 | -0.931 | -0.110 | -0.061 | 0.252 | 0.092 | 0.065 |
| Mean residuals | -0.326 | 0.554 | 0.837 | 0.673 | 0.037 | 0.139 |
| Note. Residuals of variances and covariances between variables and residuals for the mean estimates of individual variables. All residuals are standardized. VG = Video Game use and Mot = Academic motivation in writing, reading, and math. | | | | | | |

| **Table 4. Results for the traditional Cross-Lagged Panel Model (CLPM)** | | | | | | | | | | | | | | | | |  |
| --- | --- | --- | --- | --- | --- | --- | --- | --- | --- | --- | --- | --- | --- | --- | --- | --- | --- |
|  | | | | | | | | **95% Confidence Interval (β)** | | | | | | **Two-tailed** | | |  |
| **Regression path** | **Sex** | | ***B*** | | **β** | | | **Lower** | | | **Upper** | | | **p-values** | | |  |
| VG age 7 → Mot age 8 | | Boys* | -.134 |  | | -.074 |  | | -.138 |  | | -.009 |  | | .021 |  | |
|  | | Girls | .055 |  | | .020 |  | | -.036 |  | | .076 |  | | .482 |  | |
| Mot age 7 → VG age 8 | | Boys | -.017 |  | | -.039 |  | | -.102 |  | | .024 |  | | .224 |  | |
|  | | Girls | .010 |  | | .033 |  | | -.022 |  | | .088 |  | | .236 |  | |
| VG age 8 → Mot age 10 | | Boys* | -.134 |  | | -.067 |  | | -.125 |  | | -.010 |  | | .021 |  | |
|  | | Girls | .055 |  | | .017 |  | | -.030 |  | | .063 |  | | .482 |  | |
| Mot age 8 → VG age 10 | | Boys | -.017 |  | | -.040 |  | | -.104 |  | | .024 |  | | .224 |  | |
|  | | Girls | .010 |  | | .031 |  | | -.020 |  | | .081 |  | | .236 |  | |
| VG age 7 → VG age 8 | | Boys* | .259 |  | | .339 |  | | .232 |  | | .447 |  | | .000 |  | |
|  | | Girls* | .297 |  | | .364 |  | | .291 |  | | .438 |  | | .000 |  | |
| VG age 8 → VG age 10 | | Boys* | .259 |  | | .252 |  | | .147 |  | | .358 |  | | .000 |  | |
|  | | Girls* | .297 |  | | .272 |  | | .187 |  | | .357 |  | | .000 |  | |
| Mot age 7 → Mot age 8 | | Boys* | .335 |  | | .318 |  | | .258 |  | | .378 |  | | .000 |  | |
|  | | Girls* | .349 |  | | .347 |  | | .293 |  | | .400 |  | | .000 |  | |
| Mot age 8 → Mot age 10 | | Boys* | .335 |  | | .401 |  | | .329 |  | | .474 |  | | .000 |  | |
|  | | Girls* | .349 |  | | .359 |  | | .302 |  | | .416 |  | | .000 |  | |
| **Note.** Results from the Cross-Lagged panel model (CLPM) divided by sex, including cross-lagged paths, autoregressive paths, and between-person associations, respectively. *B* represents the unstandardized estimate of each regression path. β represents the standardized estimate of the regression, followed by the 95 % confidence interval of β. P-values lower than 0.05 were considered significant (marked with *). VG = Video Game playing and Mot = Academic motivation in writing, reading, and math. | | | | | | | | | | | | | | | | |  |
|  | | | | | | | | | | | | | | | | |  |

**Table 5. R (lavaan) script used to estimate models in this study**

| #Load packages  library**(**lavaan**)**  library**(**semTools**)** #Notice we use the *sem* wrapper when running our models  #Assining the dataset to a variable  dados**<-**read.csv**(**"FRQproject_obj1.5.csv", header**=TRUE**, na.strings**=**c**(**"",".","NA","-999"**))**  ##########################################################################################  #Here we create a variance/covariance matrix to test sex invariance across sexes  #This is to first check if we should specify our model separating between groups (boys and girls)  matrix **<-** "  #variances of original variables  E8_Media_VG_HQMMQ45A ~~ E8_Media_VG_HQMMQ45A  E9_Media_VG ~~ E9_Media_VG  E11_Media_VG ~~ E11_Media_VG  E8_AcadMotiv_Global_HQEET9AA ~~ E8_AcadMotiv_Global_HQEET9AA  E9_AcadMotiv_Global_IQEET9AA ~~ E9_AcadMotiv_Global_IQEET9AA  E11_AcadMotiv_Global_KQEET9AA ~~ E11_AcadMotiv_Global_KQEET9AA    #covariances  E8_Media_VG_HQMMQ45A ~~ E9_Media_VG  E8_Media_VG_HQMMQ45A ~~ E11_Media_VG  E8_Media_VG_HQMMQ45A ~~ E8_AcadMotiv_Global_HQEET9AA  E8_Media_VG_HQMMQ45A ~~ E9_AcadMotiv_Global_IQEET9AA  E8_Media_VG_HQMMQ45A ~~ E11_AcadMotiv_Global_KQEET9AA    E9_Media_VG ~~ E11_Media_VG  E9_Media_VG ~~ E8_AcadMotiv_Global_HQEET9AA  E9_Media_VG ~~ E9_AcadMotiv_Global_IQEET9AA  E9_Media_VG ~~ E11_AcadMotiv_Global_KQEET9AA    E11_Media_VG ~~ E8_AcadMotiv_Global_HQEET9AA  E11_Media_VG ~~ E9_AcadMotiv_Global_IQEET9AA  E11_Media_VG ~~ E11_AcadMotiv_Global_KQEET9AA    E8_AcadMotiv_Global_HQEET9AA ~~ E9_AcadMotiv_Global_IQEET9AA  E8_AcadMotiv_Global_HQEET9AA ~~ E11_AcadMotiv_Global_KQEET9AA    E9_AcadMotiv_Global_IQEET9AA ~~ E11_AcadMotiv_Global_KQEET9AA  "  #--  #Here we run the model freely estimated  fit_inv_free **<-** sem**(**matrix, group **=** "SEX", mimic **=** "mplus", missing **=** "ml",  data **=** dados, estimator**=**"MLR"**)**  summary**(**fit_inv_free, fit.measures**=TRUE**, standardized **=** **TRUE)**  #--  #Here we run the model constraining equality between groups (boys and girls)  fit_inv_const **<-** sem**(**matrix, group **=** "SEX", mimic **=** "mplus", missing **=** "ml",  data **=** dados, estimator**=**"MLR",  #Here we set the constrains  group.equal **=** c**(**"residual.covariances","residuals"**))**  summary**(**fit_inv_const, fit.measures**=TRUE**, standardized **=** **TRUE)**  #Compare fit of constraining for equality across groups or not  lavTestLRT**(**fit_inv_free, fit_inv_const**)**  fit_comparison_sex **<-** compareFit**(**fit_inv_free, fit_inv_const, nested **=** **TRUE)**  summary **(**fit_comparison_sex**)**  #results suggest not constraining the model between boys and girls  ##########################################################################################  #Following is the final model chosen for the study  #GROUPED RI-CLPM - freely estimated between sexes and constrained over time  #This specification was chosen after comparing fit with other competing models  model_groupDif_equalOverTime **<-** '  #Identify random intercept, i.e., individual effects  #These corresponds to avarages differences between individuals  videoGameRI =~ 1*E8_Media_VG_HQMMQ45A + 1*E9_Media_VG + 1*E11_Media_VG  motivationRI =~ 1*E8_AcadMotiv_Global_HQEET9AA + 1*E9_AcadMotiv_Global_IQEET9AA + 1*E11_AcadMotiv_Global_KQEET9AA    #Identify structured residuals  #Those are now latent variables designated by the residuals  VG1 =~ 1*E8_Media_VG_HQMMQ45A  VG2 =~ 1*E9_Media_VG  VG3 =~ 1*E11_Media_VG  Mot1 =~ 1*E8_AcadMotiv_Global_HQEET9AA  Mot2 =~ 1*E9_AcadMotiv_Global_IQEET9AA  Mot3 =~ 1*E11_AcadMotiv_Global_KQEET9AA    #Constrain variances of original variables to zero  E8_Media_VG_HQMMQ45A ~~ 0*E8_Media_VG_HQMMQ45A  E9_Media_VG ~~ 0*E9_Media_VG  E11_Media_VG ~~ 0*E11_Media_VG  E8_AcadMotiv_Global_HQEET9AA ~~ 0*E8_AcadMotiv_Global_HQEET9AA  E9_AcadMotiv_Global_IQEET9AA ~~ 0*E9_AcadMotiv_Global_IQEET9AA  E11_AcadMotiv_Global_KQEET9AA ~~ 0*E11_AcadMotiv_Global_KQEET9AA    # Regressions (AR parts + CL parts)  # On the left of each expression you tag the paths to constrain for equality across time  #Different regression coefficients for boys and girls  VG2 ~ c(ar_vg_m,ar_vg_f)*VG1 + c(mot_vg_m, mot_vg_f)*Mot1  VG3 ~ c(ar_vg_m,ar_vg_f)*VG2 + c(mot_vg_m, mot_vg_f)*Mot2  Mot2 ~ c(ar_mot_m,ar_mot_f)*Mot1 + c(vg_mot_m, vg_mot_f)*VG1  Mot3 ~ c(ar_mot_m,ar_mot_f)*Mot2 + c(vg_mot_m, vg_mot_f)*VG2      # Covariances  #In the first and second line we are specifying  #to now allow the between part to correlate with the exogenous variable  #wich would be automatic  #The first line correspond to the between effect  videoGameRI ~~ motivationRI + 0*VG1 + 0*Mot1  motivationRI ~~ 0*VG1 + 0*Mot1  #Co-movements  VG1 ~~ Mot1  VG2 ~~ Mot2  VG3 ~~ Mot3  '  #fitting freely estimated across sexes but constrined overtime  fit_groupDif_equalOverTime **<-** sem**(**model_groupDif_equalOverTime, group **=** "SEX",  missing **=** "ml", data **=** dados, estimator**=**"MLR"**)**  #Presenting summary of the model  summary**(**fit_groupDif_equalOverTime, fit.measures**=TRUE**, standardized **=** **TRUE**, rsquare**=TRUE)**  #Calculating CI of standarized estimates  standardizedsolution**(**fit_groupDif_equalOverTime, level**=**.95**)**  #Estimating local fit with standarized/normalized residuals  res **<-** residuals**(**fit_groupDif_equalOverTime, type **=** "standardized"**)**  print**(**res**)**  ##########################################################################################  #Grouped analyses  #RI-CLPM freely estimated (time and sex)  #This competing model was not chosen due to poorer fit compared to chosen model above  model_freelyEstimatedSexAndTime **<-** '  #Identify random intercept, i.e., between-person effects  #These corresponds to avarages differences between individuals  videoGameRI =~ 1*E8_Media_VG_HQMMQ45A + 1*E9_Media_VG + 1*E11_Media_VG  motivationRI =~ 1*E8_AcadMotiv_Global_HQEET9AA + 1*E9_AcadMotiv_Global_IQEET9AA + 1*E11_AcadMotiv_Global_KQEET9AA    # Identify structured residuals  #Those are now latent variables designated by the residuals  VG1 =~ 1*E8_Media_VG_HQMMQ45A  VG2 =~ 1*E9_Media_VG  VG3 =~ 1*E11_Media_VG  Mot1 =~ 1*E8_AcadMotiv_Global_HQEET9AA  Mot2 =~ 1*E9_AcadMotiv_Global_IQEET9AA  Mot3 =~ 1*E11_AcadMotiv_Global_KQEET9AA    #Constrain variances of original variables to zero  E8_Media_VG_HQMMQ45A ~~ 0*E8_Media_VG_HQMMQ45A  E9_Media_VG ~~ 0*E9_Media_VG  E11_Media_VG ~~ 0*E11_Media_VG  E8_AcadMotiv_Global_HQEET9AA ~~ 0*E8_AcadMotiv_Global_HQEET9AA  E9_AcadMotiv_Global_IQEET9AA ~~ 0*E9_AcadMotiv_Global_IQEET9AA  E11_AcadMotiv_Global_KQEET9AA ~~ 0*E11_AcadMotiv_Global_KQEET9AA    # Regressions (AR parts + CL parts)  VG2 ~ VG1 + Mot1  VG3 ~ VG2 + Mot2  Mot2 ~ Mot1 + VG1  Mot3 ~ Mot2 + VG2    #Covariances  #In the first and second line we are saying  #to now allow the between part to correlate with the exogenous variable  #The first line correspond to the between effect  videoGameRI ~~ motivationRI + 0*Mot1 + 0*VG1  motivationRI ~~ 0*VG1 + 0*Mot1  #Co-movements  VG1 ~~ Mot1  VG2 ~~ Mot2  VG3 ~~ Mot3  '  #fitting freely estimated across sexes and time  fit_free **<-** sem**(**model_freelyEstimatedSexAndTime, group **=** "SEX", missing **=** "ml",  data **=** dados, estimator**=**"MLR"**)**  summary**(**fit_free, fit.measures**=TRUE**, standardized **=** **TRUE**, rsquare**=TRUE)**  #------  #Compare fit of the two competing models: one with time constrains and the other without  compareFit_timeConstrains **<-** compareFit**(**fit_free, fit_groupDif_equalOverTime, nested **=** **TRUE)**  summary **(**compareFit_timeConstrains**)**  #===========================================================================================  #Traditional CLPM for sensitivity analysis  #TRADITIONAL CLPM GROUPED (BOYS AND GIRLS)  #Constrained over time  CLPMModel_Grouped_contrained_overtime **<-** '    # Regressions (AR parts + CL parts)  # On the left of each expression we tag the paths to constrain for equality  E9_Media_VG ~ c(ar_vg_m,ar_vg_f)*E8_Media_VG_HQMMQ45A + c(mot_vg_m, mot_vg_f)*E8_AcadMotiv_Global_HQEET9AA  E11_Media_VG ~ c(ar_vg_m,ar_vg_f)*E9_Media_VG + c(mot_vg_m, mot_vg_f)*E9_AcadMotiv_Global_IQEET9AA    E9_AcadMotiv_Global_IQEET9AA ~ c(ar_mot_m,ar_mot_f)*E8_AcadMotiv_Global_HQEET9AA + c(vg_mot_m, vg_mot_f)*E8_Media_VG_HQMMQ45A  E11_AcadMotiv_Global_KQEET9AA ~ c(ar_mot_m,ar_mot_f)*E9_AcadMotiv_Global_IQEET9AA + c(vg_mot_m, vg_mot_f)*E9_Media_VG    #Covariances  #Co-movements  E8_Media_VG_HQMMQ45A ~~ E8_AcadMotiv_Global_HQEET9AA  E9_Media_VG ~~ E9_AcadMotiv_Global_IQEET9AA  E11_Media_VG ~~ E11_AcadMotiv_Global_KQEET9AA  '  #fitting freely estimated across sexes but not time  fit_groupedCLPM2 **<-** sem**(**CLPMModel_Grouped_contrained_overtime, missing **=** "ml", group **=** "SEX",  data **=** dados, estimator**=**"MLR"**)**  summary**(**fit_groupedCLPM2, fit.measures**=TRUE**, standardized **=** **TRUE)**  #Standarized CI  standardizedsolution**(**fit_groupedCLPM2, level**=**.95**)**  #Testing differences between the traditional CLPM and the RI-CLPM  lavTestLRT**(**fit_groupDif_equalOverTime, fit_groupedCLPM2**)** |
| --- |
